# Tang-Nai-Kang Alleviates Pre-diabetes and Metabolic Disorders and Induces a Gene Expression Switch toward Fatty Acid Oxidation in SHR.Cg-Leprcp/NDmcr Rats

**DOI:** 10.1371/journal.pone.0122024

**Published:** 2015-04-13

**Authors:** Linyi Li, Hisae Yoshitomi, Ying Wei, Lingling Qin, Jingxin Zhou, Tunhai Xu, Xinli Wu, Tian Zhou, Wen Sun, Xiangyu Guo, Lili Wu, Haiyan Wang, Yan Zhang, Chunna Li, Tonghua Liu, Ming Gao

**Affiliations:** 1 Health-cultivation Laboratory of the Ministry of Education, Beijing University of Chinese Medicine, Beijing, China; 2 School of Pharmaceutical Sciences, Mukogawa Women’s University, Hyogo, Japan; 3 Dongzhimen Hospital Eastern Affiliated to Beijing University of Chinese Medicine, Beijing, China; 4 Dongfang Hospital Affiliated to Beijing University of Chinese Medicine, Beijing, China; East Tennessee State University, UNITED STATES

## Abstract

Increased energy intake and reduced physical activity can lead to obesity, diabetes and metabolic syndrome. Transcriptional modulation of metabolic networks has become a focus of current drug discovery research into the prevention and treatment of metabolic disorders associated with energy surplus and obesity. Tang-Nai-Kang (TNK), a mixture of five herbal plant extracts, has been shown to improve abnormal glucose metabolism in patients with pre-diabetes. Here, we report the metabolic phenotype of SHR.Cg-*Lepr* ^cp^/NDmcr (SHR/cp) rats treated with TNK. Pre-diabetic SHR/cp rats were randomly divided into control, TNK low-dose (1.67 g/kg) and TNK high-dose (3.24 g/kg) groups. After high-dose treatment for 2 weeks, the serum triglycerides and free fatty acids in SHR/cp rats were markedly reduced compared to controls. After 3 weeks of administration, the high dose of TNK significantly reduced the body weight and fat mass of SHR/cp rats without affecting food consumption. Serum fasting glucose and insulin levels in the TNK-treated groups decreased after 6 weeks of treatment. Furthermore, TNK-treated rats exhibited obvious improvements in glucose intolerance and insulin resistance. The improved glucose metabolism may be caused by the substantial reduction in serum lipids and body weight observed in SHR/cp rats starting at 3 weeks of TNK treatment. The mRNA expression of NAD^+^-dependent deacetylase sirtuin 1 (SIRT1) and genes related to fatty acid oxidation was markedly up-regulated in the muscle, liver and adipose tissue after TNK treatment. Furthermore, TNK promoted the deacetylation of two well-established SIRT1 targets, PPARγ coactivator 1α (PGC1α) and forkhead transcription factor 1 (FOXO1), and induced the phosphorylation of AMP-activated protein kinase (AMPK) and acetyl-CoA carboxylase (ACC) in different tissues. These observations suggested that TNK may be an alternative treatment for pre-diabetes and metabolic syndrome by inducing a gene expression switch toward fat oxidation through the activation of SIRT1 and AMPK signaling.

## Introduction

Pre-diabetes is a condition in which plasma glucose is elevated above the normal range but below that of clinical diabetes and includes impaired fasting glucose (IFG) or glucose intolerance (IGT). Both IFG and IGT are high-risk states for type 2 diabetes and cardiovascular diseases [1–3]. Approximately three-fourths of individuals with both IFG and IGT have metabolic syndrome (MetS) [4], which is characterized by dyslipidemia, disorders of glucose metabolism, hypertension and obesity. Pre-diabetes is somewhat predictive of macrovascular diseases, but most of this association seems to be mediated through MetS [4–6]. Therefore, MetS can be considered a pre-diabetic state [4,7].

Lifestyle interventions are considered requisites to combat metabolic diseases related to energy surplus, such as obesity and diabetes. Metformin and troglitazone can significantly reduce the risk of diabetes [8,9], and metformin has been recommended as a pharmacological therapy for individuals with IFG/IGT by the American Diabetes Association since 2007. These studies provide insight into preventing pre-diabetes effectively. However, lifestyle interventions are not usually successful as sustained effort required for diet control and physical exercise causes stress to patients. Moreover, the increased risk of gastrointestinal symptoms, cognitive impairment [10] and lactic acidosis caused by metformin and the risk of obesity and liver toxicity associated with troglitazone also cannot be neglected. Therefore, a more efficient agent is needed to prevent and treat pre-diabetes and metabolic disorders. In the field of metabolism regulation, intense drug discovery efforts are currently focused on promoting energy consumption in organs that specialize in energy expenditure.

An organism can adapt to nutrient availability through the transcriptional modulation of metabolic networks [11,12]. Sirtuins are a family of NAD^+^-dependent deacetylases that sense cellular nutrient status and modulate gene expression through the deacetylation of N-acetyl lysines on various protein substrates, including transcription factors and coregulators [13–15]. Sirtuin 1 (SIRT1) can respond to energy restriction and promote longevity by regulating a wide range of biological processes, including fat oxidation, lipolysis and mitochondrial energy metabolism, by deacetylating PPARγ coactivator 1α (PGC1α) and forkhead transcription factor (FOXO) 1 [15–20]. AMP-activated protein kinase (AMPK) is another primary energy sensor that helps to maintain energy homeostasis in the whole body [21,22]. In skeletal and cardiac muscle, AMPK activation leads to phosphorylation of acetyl coenzyme A carboxylase (ACC), subsequently reducing the concentration of malonyl coenzyme A, an inhibitor of carnitine palmitoyltransferase (CPT); these effects, in turn, enhance fatty acid oxidation by accelerating CPT-mediated fatty acid translocation into mitochondria [23–25]. AMPK can also regulate the activity of transcriptional factors and coregulators related to fatty acid oxidation and mitochondrial function, such as PGC1α, peroxisome proliferator-activated receptor (PPAR) α and PPARβ/δ [26–28]. Both SIRT1 and AMPK have been considered as attractive targets for the regulation of transcriptional networks to promote whole-body energy expenditure and treat metabolic disorders. Recent reports using different transgenic models have suggested that SIRT1 and AMPK might act in an orchestrated signaling network to improve metabolic disorders [29,30]. The natural calorie restriction-mimetic compound resveratrol and synthetic SIRT1 activators protect against diet-induced metabolic disorders by activating both SIRT1 and AMPK [31,32]. Therefore, pharmacological interventions that target the SIRT1-AMPK network and regulate global gene expression programs that promote energy expenditure may open new avenues for the treatment of energy surplus-related disorders.

For thousands of years, traditional herbal medicines have played an important role in health maintenance for people throughout the world. Multi-herbal formulas can effectively ameliorate insulin resistance syndrome and diabetes [33–36]. Tang-Nai-Kang (TNK) is a mixture of extracts from five herbal plants: *Fructus Ligustri Lucidi* (Oleaceae), *Spica Prunellae Vulgaris* (Labiatae), *Saururus chinensis* (Saururaceae), *Psidium guajava* (Myrtaceae) and *Radix Ginseng* (Araliaceae). Over the years, these herbs have been widely used to treat diabetes mellitus, and their anti-diabetic activities have been confirmed by more-recent research. It has been reported that *Spica Prunellae Vulgaris* and *Saururus chinensis* both possess anti-diabetic, anti-inflammatory, hepatoprotective and hypotensive effects *in vivo* and *in vitro*, and the fruits of *Fructus Ligustri Lucidi* decrease the blood glucose level in diabetic rats [37–39]. The leaves of *Psidium guajava* have been reported to have hypoglycemic, hypolipidemic, hepatoprotective and anti-obesity effects in various animal models [40]. Many studies have suggested that *Panax ginseng* improves glucose and lipid metabolism, prevents oxidative stress, and ameliorates insulin resistance and obesity in diabetic patients and animal models [41,42]. TNK has been effective in improving abnormal glucose metabolism in patients with IGT and IFG [43]. TNK can reduce fasting blood glucose (FBG) and lipid levels, increase the insulin sensitivity index, and ameliorate pathological changes in the pancreas in Zucker fatty rats [44]. However, there have inadequate pharmacological and mechanistic studies of the effects of TNK on pre-diabetes and MetS. Investigating the metabolic effects and the mechanism of TNK on pre-diabetes and MetS has practical applications for preventing or delaying insulin resistance, IGT and possibly the development of clinical diabetes and MetS.

Leptin receptor-deficient SHR.Cg-*Lepr* ^cp^/NDmcr (SHR/cp) rats spontaneously develop moderately elevated blood glucose with hyperphagia, insulin resistance, obesity, dyslipidemia and hypertension, traits that resemble those of human pre-diabetes and MetS [45,46]. In the present study, we investigated the potential beneficial effects and mechanisms of TNK in SHR/cp rats. Our results demonstrated that TNK administration improved pre-diabetes and MetS by inducing gene expression involved in fatty acid oxidation in white adipose tissue (WAT), skeletal muscle and liver through the activation of SIRT1 and AMPK signaling in SHR/cp rats.

## Materials and Methods

### Preparation of TNK

TNK contains *Fructus Ligustri Lucidi* (Oleaceae), *Spica Prunellae Vulgaris* (Labiatae), *Herba Saururi* (Saururaceae), *Psidium guajava* (Myrtaceae) and *Radix Ginseng* (Araliaceae), in a proportion of 4:4:2:2:1 [47]. The preparation of TNK was performed as previously described [44,47]. The first four plants were mixed and extracted twice with refluxing 75% (v/v) ethanol (1:8 w/v) for 1 hr; then, the obtained solution was concentrated into an ethanol extract with a recovery rate of 13.83%. Afterwards, the obtained residue was decocted twice with water (1:6 w/v) for 1 hr, and the obtained solution was concentrated into a water extract with a recovery rate of 5.49%. To dry the *Radix Ginseng*, the plants were baked at 60°C for 4 hrs, and the dried *Radix Ginseng* was crushed to yield a fine powder (150 ± 6.6 μm). Last, all components were mixed in a homogenous manner according to the following weight ratio: ethanol extract/water extract/*Radix Ginseng* fine power/excipients = 25/10/15/10 [47]. The entire process was carried out by Sichuan Medco Pharmaceutical Limited Corporation (Deyang, China). All crude drugs were purchased from Shenyang Pharmaceutical Group Corporation (Shenyang, China) and authenticated following the pharmacopoeia of the People’s Republic of China (2000 ed). In the spectrophotometry analysis, the proportions of total flavonoids and triterpenoid saponin in the ethanol extract were 4.23% and 3.67%, respectively, while for the water extract, the total polysaccharide content was 5.83% (S1 Text and S1 Table). The concentrations of Rg1, Re, Rb1 and rosmarinic acid in the TNK were determined to be 0.09%, 0.12%, 0.10%, and 0.55%, respectively, using high-performance liquid chromatography (HPLC) analysis (S2 Text and S1–S3 Figs).

### Animals

The SHR/cp rats (Japan SLC, Inc., Shizuoka, Japan) were 7-week-old males with body weights of 190–210 g. The Wistar Kyoto (WKY) rats (Japan SLC, Inc., Shizuoka, Japan) were 7-week-old males with body weights of 150–170 g. Animals were kept in specific-pathogen-free (SPF) animal rooms at Mukogawa Women's University and maintained at a temperature of 22–24°C and 40–60% humidity with free access to normal chow and water throughout the experiment. This study was conducted in accordance with the Guidelines for the Care and Use of Laboratory Animals of Mukogawa Women's University. All animal protocols were approved by the Animal Care and Use Committee of Mukogawa Women's University.

SHR/cp rats, which have pre-diabetes and MetS, were chosen according to the levels of FBG, total triglycerides (TG) and total cholesterol (TC) in the serum, body weight and systolic blood pressure (SBP) (Table 1). The rats were randomly divided into three groups (n = 7): (1) rats treated with 1.67 g/kg TNK (TL), (2) rats treated with 3.24 g/kg TNK (TH) and (3) an untreated control group (CON). TNK was prepared in sterile water and administered once daily by gastric gavage for 7 consecutive weeks. The CON group received the same volume of sterile water. Age-matched male WKY rats were used as normal controls.

**Table 1:** SHR-cp rats used in the study.

**Group**	**Dose (g/kg)**	**FBG (mg/dL)**	**Body weight (g)**	**TG (mg/dL)**	**TC (mg/dL)**	**SBP (mmHg)**
**WKY**	**–**	123.7±11.3***	157.1±8.2***	59.7±9.8***	95.0±11.5	115.5±12.5***
**CON**	**–**	162.1±12.6	206.6±8.5	140.5±26.1	95.8±16.0	142.1±9.8
**TL**	1.67	162.7±12.1	208.7±5.9	143.3±17.8	90.1±8.4	144.9±13.9
**TH**	3.24	160.8±12.4	206.6±7.7	140.1±34.6	89.4±14.3	147.6±7.4

Data are expressed as the mean ± SD.

***P < 0.001 vs. CON. TNK, Tang-Nai-Kang; SHR/cp, SHR.Cg-*Lepr* ^cp^/NDmcr rat; WKY, Wistar Kyoto rat; CON, control group; TL, low-dose TNK group (1.67 g/kg); TH, high-dose TNK group (3.24 g/kg); FBG, fasting blood glucose; TG, total triglycerides; TC, total cholesterol; FFA, free fatty acids; SBP, systolic blood pressure.

### Analysis of body weight, food intake and SBP

Body weight and food intake were recorded every three days throughout the experiment. SBP was determined by the indirect tail-cuff method using a blood pressure monitor (Softron, Tokyo, Japan).

### Biochemical analysis

FBG, TG, TC, aspartate aminotransferase (AST) and alanine aminotransferase (ALT) were measured using commercially available kits (Wako Pure Chemical Industries, Osaka, Japan). Fasting serum insulin (FINS) was determined using enzyme-linked immunosorbent assay (ELISA) kits (ALPCO, Salem, NH) as instructed by the manufacturers. Free fatty acids (FFA) were measured using commercially available kits (Wako Pure Chemical Industries). Insulin sensitivity was assessed using the homeostasis model of assessment-insulin resistance index (HOMA-IR), which was calculated using the following equation: HOMA-IR = FPG (mM) × FINS (ng/mL)/22.5 [48]. A protein content assay was carried out according to the method of Lowry [49].

### Oral glucose tolerance test (OGTT) and insulin tolerance test (ITT)

Blood was sampled via the tail vein from rats after 12 hrs of fasting. FBG was quantified using the glucose oxidase method at 0, 30, 60 and 120 min after glucose (2 g/kg) loading in the OGTT and at 40 and 90 min after subcutaneous injection of insulin (0.4 IU/kg) in the ITT. The area under the curve (AUC) was then calculated from the serum glucose content.

### Assessment of body composition

Body composition was analyzed by bioelectrical impedance analysis (BIA) (ImpediVet, ImpediMed Ltd., Brisbane, Australia) as previously described [50]. Acquired data were downloaded and processed using bioimpedance software (ImpediVet Vet BIS1 v. 1.0.2).

### Histological examination of adipose tissue

After animals were sacrificed, epididymal adipose tissues were isolated, washed with normal saline, wiped dry, fixed in paraformaldehyde, conventionally embedded in paraffin, sectioned (5 μm thick), stained with hematoxylin-eosin (HE) and examined under an optical microscope (Olympus CX41RF, Olympus, Tokyo, Japan).

### Liver histopathology and lipid determination

Following treatment, the livers were excised and fixed in 4% paraformaldehyde. They were then paraffin embedded and sectioned at a thickness of 3 μm. Tissue sections were routinely stained with HE and examined under an optical microscope (Olympus CX41RF, Olympus, Tokyo, Japan). For the measurement of liver lipids, a 50-mg aliquot of liver was homogenized, and the lipids were extracted. The levels of TG in the liver tissues were determined using a serum TG determination kit (BioSino, Inc., China).

### Quantitative real-time PCR

Liver and muscle tissue samples (approximately 100 mg) and adipose tissue samples (approximately 300 mg) were removed from -80°C to prepare RNA samples. RNA was then reverse-transcribed into cDNA (TOYOBO, Tokyo, Japan), diluted in RNase-free water to a final concentration of 20 ng/μL, and used for amplification of target genes in real-time PCR with THUNDERBIRD SYBR qPCR Mix (TOYOBO, Tokyo, Japan). The specific primers (S2 Table) were designed from sequences available from GenBank and were synthesized by Invitrogen (Beijing, China). The amplification was performed as follows in a real-time PCR system (ABI Prism 7500): 1 cycle of 95°C for 30 s and 40 cycles of 95°C for 5 s and 60°C for 30 s. The fold differences in mRNA expression levels between samples were calculated using the 2^-ΔΔ Ct^ relative quantification method.

### Western blot analysis

Liver and muscle tissue samples (approximately 200 mg) and adipose tissue samples (approximately 1 g) were removed from -80°C storage. In total, 600 μL of protein lysis buffer containing phosphatase inhibitors and protease inhibitors was added, and lysates were sonicated until lysis was complete. Samples were placed in an ice bath for 35 min and mixed thoroughly at appropriate times. Samples were centrifuged at 12,000 rpm for 10 min. The supernatant was collected to obtain the total protein samples. The supernatant was mixed thoroughly with sample buffer, boiled, and submitted to SDS-PAGE analysis. The immunoblotting procedure was performed as previously described [51]. The acetylation levels of PGC1α and FOXO1 were determined by immunoprecipitating total protein and immunoblotting with an antibody raised against acetylated lysine. The following antibodies were used: anti-AMPK α, ACC, P-AMPKα, P-ACC, PGC1α, FOXO1, acetylated lysine and β-actin antibodies (Cell Signaling Technology, MA, USA). Protein band densities were analyzed using Gel-Pro Analyzer 3.1 software.

### Statistical analysis

Data are expressed as the mean ± the standard deviation of the mean (SD). Data were analyzed by one-way analysis of variance with Bonferroni's correction for multiple pairwise comparisons. Significant differences were deemed significant if P < 0.05.

## Results

### TNK ameliorates pre-diabetes and MetS in SHR/cp rats

**Fig 1:**
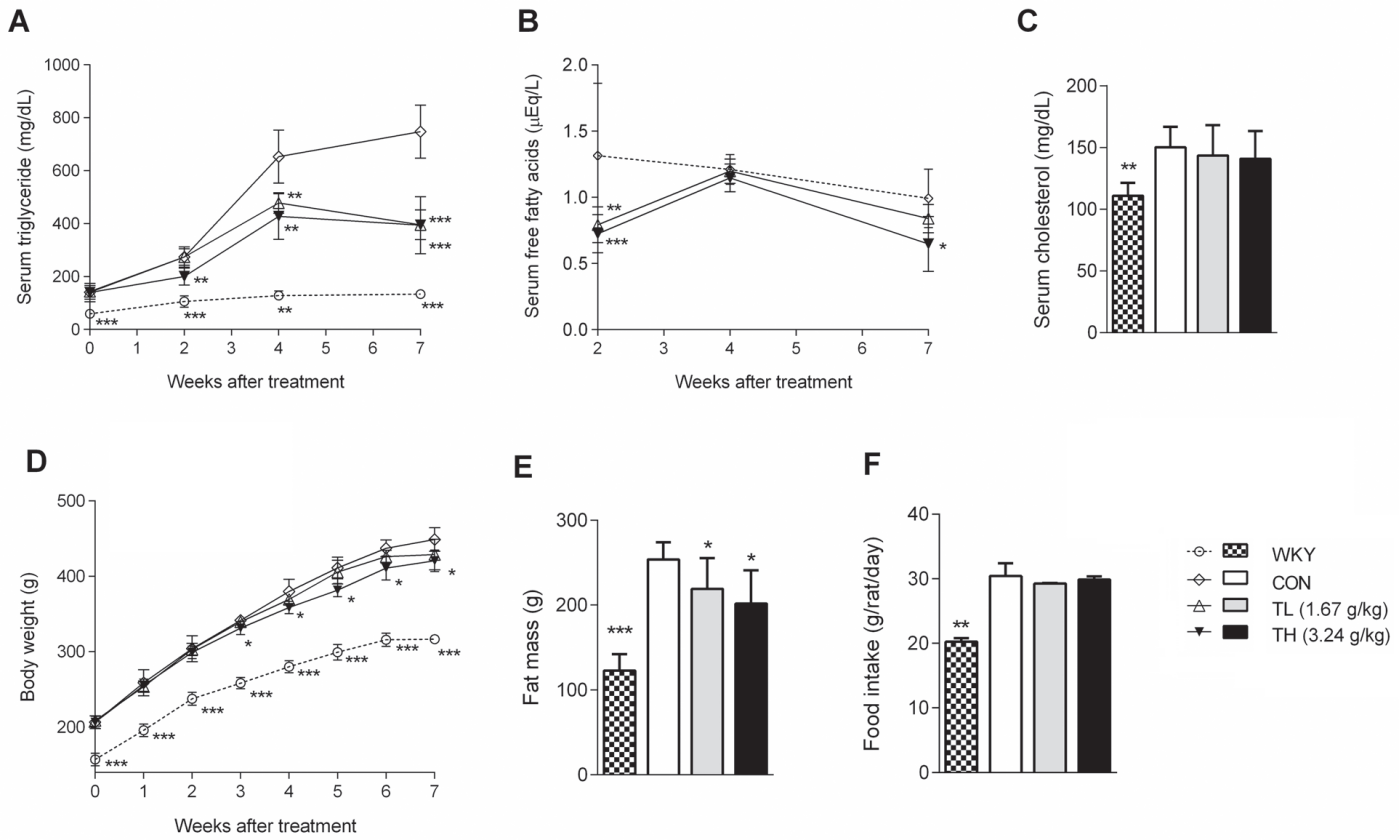
TNK improves hyperlipidemia and obesity in SHR/cp rats. (A) Serum fasting TG, (B) FFA and (C) TC levels in SHR/cp rats treated with 1.67 or 3.24 g/kg TNK for 7 weeks (n = 7). (D) Body weight evolution of SHR/cp rats treated with 1.67 or 3.24 g/kg TNK or untreated controls (n = 7). (E) Fat mass (n = 7). (F) Average food consumption (n = 7). Data are shown as the mean ± SD. *P < 0.05, **P < 0.01, ***P < 0.001 vs. CON. TNK, Tang-Nai-Kang; SHR/cp, SHR.Cg-*Lepr* ^cp^/NDmcr rat; WKY, Wistar Kyoto rat; CON, control group; TL, low-dose TNK group (1.67 g/kg); TH, high-dose TNK group (3.24 g/kg); TG, total triglycerides; TC, total cholesterol; FFA, free fatty acids.

**Fig 2:**
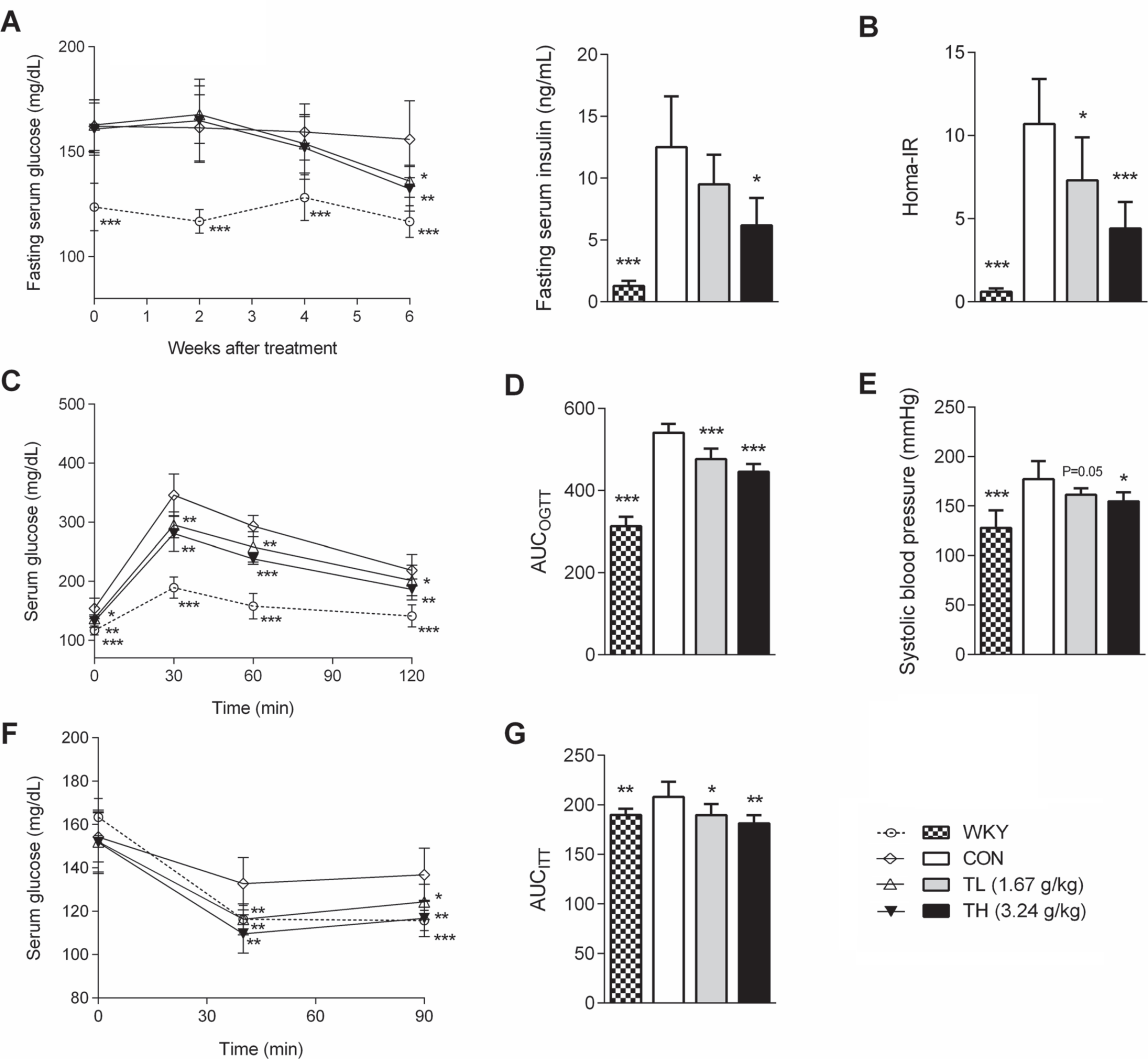
TNK improves glucose homeostasis and insulin resistance in SHR/cp rats. (A) FBG and FINS levels in SHR/cp rats treated with 1.67 or 3.24 g/kg TNK for 7 weeks. (B) HOMA-IR. (C) Serum glucose curve and (D) area under the blood glucose curve (AUC) in OGTT on SHR/cp rats treated with TNK for 6 weeks and orally administered 2 g/kg glucose. (E) SBP of SHR/cp rats treated with 1.67 or 3.24 g/kg TNK for 6 weeks. (F) Serum glucose curve and (G) area under the blood glucose curve (AUC) in ITT on SHR/cp rats treated with TNK for 5 weeks and injected with 0.4 IU /kg insulin after 6 hrs of fasting. Data are shown as the mean ± SD. *P < 0.05, **P < 0.01, ***P < 0.001 vs. CON. (n = 7). TNK, Tang-Nai-Kang; SHR/cp, SHR.Cg-*Lepr* ^cp^/NDmcr rat; WKY, Wistar Kyoto rat; CON, control group; TL, low-dose TNK group (1.67 g/kg); TH, high-dose TNK group (3.24 g/kg); FBG, fasting blood glucose; FINS, fasting serum insulin; OGTT, oral glucose tolerance test; ITT, insulin tolerance test; SBP, systolic blood pressure; HOMA-IR, homeostasis model of assessment-insulin resistance index; AUC, area under the curve.

As shown in Table 1 and Figs 1 and 2, obese SHR/cp rats exhibited modestly but significantly elevated FBG and much higher insulin than WKY rats. Meanwhile, SHR/cp rats showed higher serum TG and SBP compared to WKY rats. The OGTT and ITT showed glucose intolerance and insulin insensitivity in SHR/cp rats, demonstrating their impaired glucose/lipid metabolism, hypertension and systemic insulin resistance.

Following the 2-week treatment with TNK, the serum TG and FFA levels in SHR/cp rats were markedly reduced by 33% and 32%, respectively, compared with the controls (Fig 1A and 1B). The low dose of TNK slightly, but not significantly, reduced body weight in SHR/cp rats, while the high-dose treatment significantly reduced body weight and fat mass after 3 weeks of administration without affecting the amount of food consumption (Fig 1D, 1E and 1F). The FBG and insulin levels in the TNK-treated groups decreased after 7 weeks of treatment, with a marked reduction in the HOMA-IR index (Fig 2A and 2B). Furthermore, the OGTT and ITT showed improved glucose metabolism and a better response of fasting serum glucose to insulin (Fig 2C, 2D, 2F and 2G). Moreover, the high dose of TNK significantly reduced the SBP of SHR/cp rats (Fig 2E). Altogether, these results demonstrate that TNK improved hyperlipidemia, obesity, IFG, IGT, insulin sensitivity and hypertension in SHR/cp rats.

### TNK induces a gene expression switch toward fatty acid oxidation in metabolic tissues

**Fig 3:**
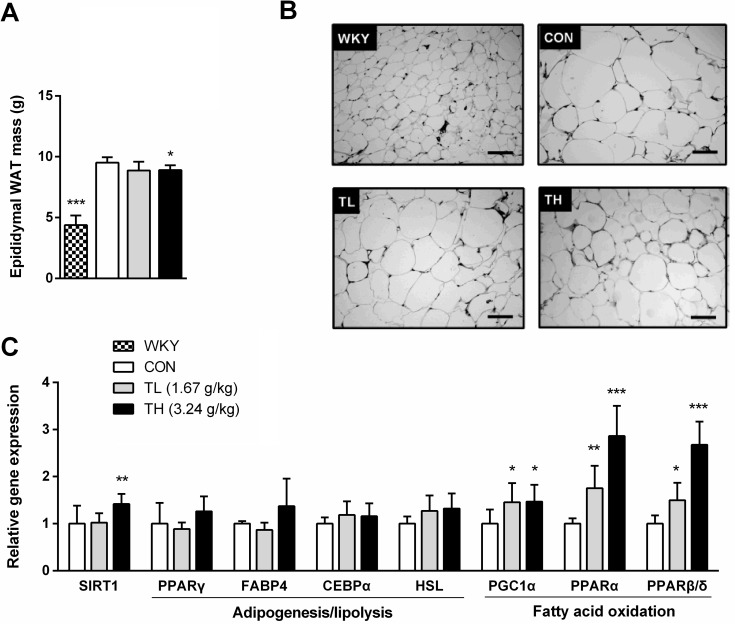
TNK reduces fat storage in the adipose tissue of SHR/cp rats. (A) E-WAT mass was measured in SHR/cp rats treated with 1.67 or 3.24 g/kg TNK for 7 weeks (n = 6). (B) Representative HE staining (200×) in E-WAT. (C) Gene expression profile in the E-WAT. Values are expressed relative to β-actin (n = 5). Data are shown as the mean ± SD. *P < 0.05, **P < 0.01, ***P < 0.001 vs. CON. TNK, Tang-Nai-Kang; SHR/cp, SHR.Cg-*Lepr* ^cp^/NDmcr rat; WKY, Wistar Kyoto rat; CON, control group; TL, low-dose TNK group (1.67 g/kg); TH, high-dose TNK group (3.24 g/kg); E-WAT, epididymal white adipose tissue; SIRT1, sirtuin 1; CEBPα, CCAAT/enhancer-binding protein α; FABP4, fatty acid-binding protein 4; HSL, hormone-sensitive lipase; PGC1α, peroxisome proliferator activated receptor-γ coactivator 1α; PPARα, PPARβ/δ, PPARγ, peroxisome proliferator activated receptor-α, -β/δ, -γ. HE, hematoxylin-eosin.

Because TNK treatment did not affect food intake, the reduction in body adiposity and insulin resistance of SHR/cp rats might be attributed to the effects of TNK on energy expenditure. After a 7-week treatment with TNK, the epididymal WAT mass and adipocyte size were significantly reduced compared to controls (Fig 3A and 3B) and the mRNA expression levels of fatty acid oxidative markers, PGC1α and the nuclear receptors PPARα and PPARβ/δ were elevated, suggesting that TNK might promote energy expenditure by enhancing fatty acid oxidation in WAT of SHR/cp rats (Fig 3C). Importantly, it seems that these effects of TNK did not result from damage to adipocyte differentiation and lipid storage capacity, as PPARγ expression was slightly elevated, and its downstream targets CEBPα and FABP4 were not significantly affected. In addition, hormone-sensitive lipase (HSL) expression was unchanged compared to controls (Fig 3C).

**Fig 4:**
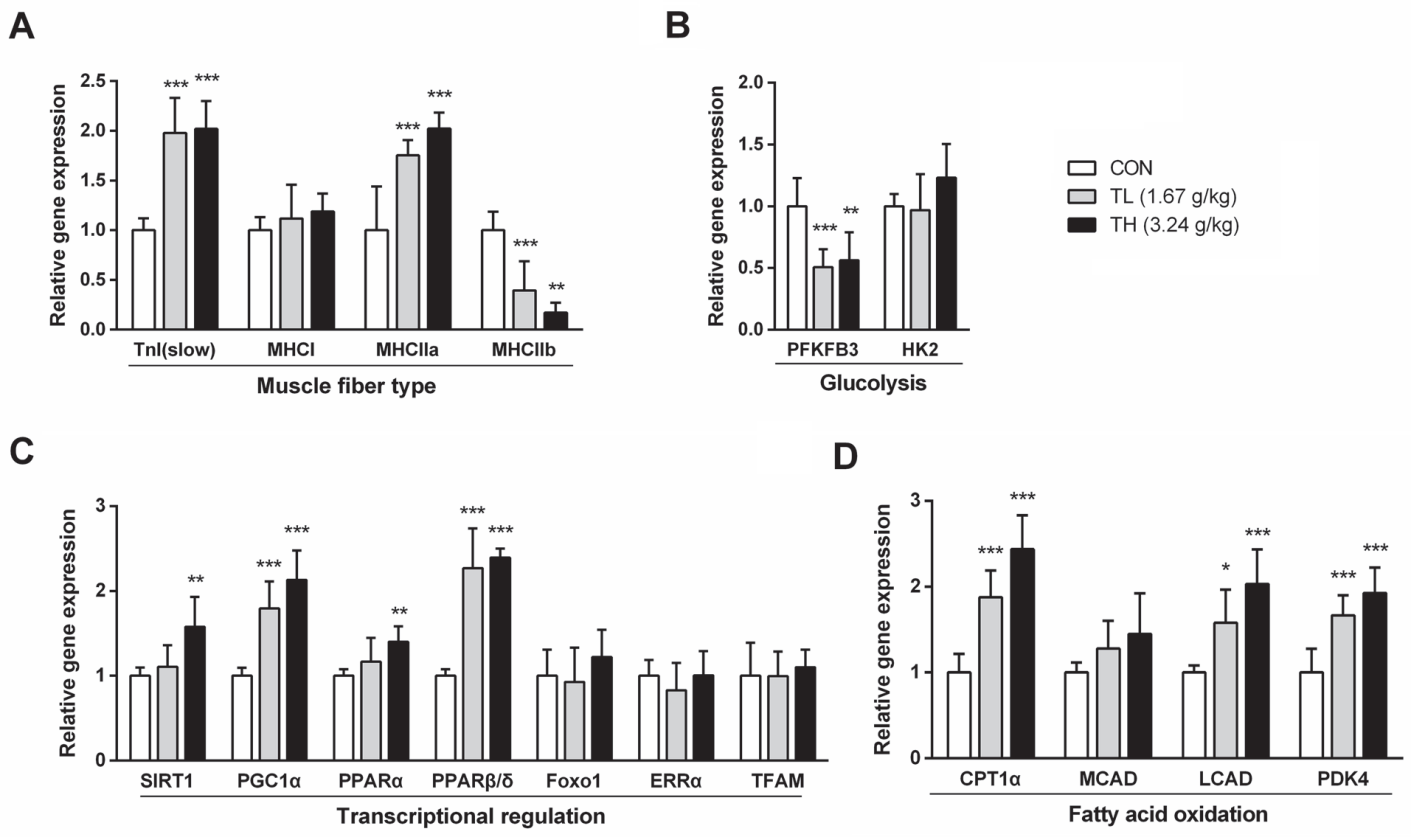
TNK enhances fat oxidation-related gene expression in the muscle of SHR/cp rats. The expression of genes related to muscle fiber type (A), glycolysis (B), transcriptional regulation (C) and fatty acid oxidation (D) in skeletal muscle relative to β-actin. (n = 5). Data are shown as the mean ± SD. *P < 0.05, **P < 0.01, ***P < 0.001 vs. CON. TNK, Tang-Nai-Kang; SHR/cp, SHR.Cg-*Lepr* ^cp^/NDmcr rat; WKY, Wistar Kyoto rat; CON, control group; TL, low-dose TNK group (1.67 g/kg); TH, high-dose TNK group (3.24 g/kg); CEBPα, CCAAT/enhancer-binding protein α; CPT1α, carnitine palmitoyltransferase 1α; ERRα, estrogen-related receptor α; FOXO1, forkhead transcription factor 1; HK2, hexokinase 2; LCAD, long-chain-acyl-CoA dehydrogenase; MHCI, MHCIIa, MHCIIb, myosin heavy chains, type I, IIa, IIb; PDK4, pyruvate dehydrogenase kinase 4; PFKFB3, 6-phosphofructo-2-kinase/fructose-2, 6-biphosphatase 3; PGC1α, peroxisome proliferator activated receptor-γ coactivator 1α; PPARα, PPARβ/δ, peroxisome proliferator activated receptor-α, -β/δ; SIRT1, sirtuin 1; TFAM, mitochondrial transcription factor; TnI (slow), slow-twitch skeletal muscle troponin I.

We further investigated the expression levels of genes related to energy expenditure in skeletal muscle, which is one of the major energy-dissipating organs. The mRNA expression levels of slow-twitch fiber markers, such as slow twitch skeletal muscle troponin I (TnI_slow_) and myosin type IIa heavy chains (MHCIIa), were significantly increased, together with the reduced expression of fast-twitch glycolytic type IIb (MHCIIb) in skeletal muscle, suggesting that TNK treatment might enhance the oxidative capacity of skeletal muscle fiber in SHR/cp rats (Fig 4A). Moreover, this switch was associated with reduced mRNA expression of 6-phosphofructo-2-kinase/fructose-2, 6-biphosphatase 3 (PFKFB3) (Fig 4B), a glycolytic enzyme that is central to glycolytic flux, and with elevated mRNA expression of pyruvate dehydrogenase kinase-4 (PDK4), suggesting that TNK could promote a switch in the energy source from glucose to fatty acids by reducing pyruvate utilization. TNK also elevated the mRNA expression of PGC1α, PPARα, PPARβ/δ, CPT1α and long-chain-acyl-CoA dehydrogenase (LCAD), which promote fatty acid oxidation (Fig 4C and 4D). Therefore, TNK might drive the increase in the oxidative capacity of skeletal muscle fiber by inducing a gene expression switch toward fatty acid oxidation in the skeletal muscle of SHR/cp rats.

**Fig 5:**
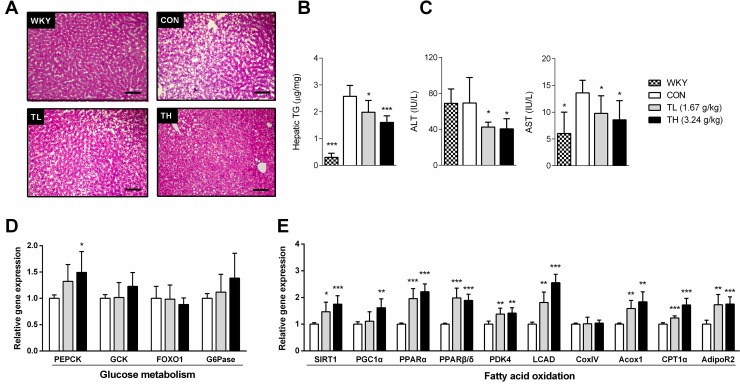
TNK improves fatty liver and promotes hepatic fatty oxidation-related gene expression in SHR/cp rats. (A) Representative HE staining (200×) of liver sections from SHR/cp rats treated with 1.67 or 3.24 g/kg TNK for 7 weeks. (B) Hepatic TG content (n = 6). (C) ALT and AST were measured in the sera of SHR/cp rats treated with 1.67 or 3.24 g/kg TNK for 7 weeks (n = 6). The expression of genes related to glucose metabolism (D) and fatty acid oxidation (E) in the liver relative to β-actin (n = 5). Data are shown as the mean ± SD. *P < 0.05, **P < 0.01, ***P < 0.001 vs. CON. TNK, Tang-Nai-Kang; SHR/cp, SHR.Cg-*Lepr* ^cp^/NDmcr rat; WKY, Wistar Kyoto rat; CON, control group; TL, low-dose TNK group (1.67 g/kg); TH, high-dose TNK group (3.24 g/kg); AST, aspartate aminotransferase; ALT, alanine aminotransferase; TG, total triglycerides; Acox1, acetyl-CoA carboxylase 1; AdipoR2, adiponectin receptor 2; CPT1α, carnitine palmitoyltransferase 1α; CoxIV, cytochrome c oxidase subunit IV; FOXO1, forkhead transcription factor 1; G6Pase, glucose-6-phosphatase; GCK, glucokinase; LCAD, long-chain-acyl-CoA dehydrogenase; PDK4, pyruvate dehydrogenase kinase 4; PEPCK, phosphoenolpyruvate carboxykinase; PGC1α, peroxisome proliferator activated receptor-γ coactivator 1α; PPARα, PPARβ/δ, peroxisome proliferator activated receptor-α, -β/δ; SIRT1, sirtuin 1.

We also investigated the lipid metabolism and gene expression related to energy expenditure in the liver tissue of SHR/cp rats. The SHR/cp rats developed marked hepatic steatosis, which was visualized by HE staining, showing large areas of microvesicular fat droplets and macrovesicular hepatocellular vacuolation. After the 7-week treatment with TNK, reduced areas of hepatocellular vacuolation and triglyceride content suggested that hepatic storage of lipid droplets was significantly decreased compared to controls (Fig 5A and 5B). The reduced serum ALT and AST further demonstrated that TNK improved fatty liver in SHR/cp rats (Fig 5C). We then tested whether TNK could promote energy expenditure-related gene expression in the liver of SHR/cp rats. The expression of phosphoenolpyruvate carboxykinase (PEPCK), a critical kinase enzyme that controls gluconeogenesis, was modestly increased, while glucose-6-phosphatase (G6Pase), another critical enzyme related to gluconeogenesis, remained unchanged (Fig 5D). However, TNK strongly induced the expression of enzymes and regulators that control fatty acid oxidation in the liver of SHR/cp rats, as shown by the significantly induced mRNA expression of two nuclear receptors, PPARα and PPARβ/δ, in the liver of TNK-treated SHR/cp rats. The PPAR target genes PDK4, acetyl-CoA carboxylase (Acox) 1 and adiponectin receptor (AdipoR) 2 were consistently elevated (Fig 5E). These results suggest that TNK might promote hepatic energy expenditure by primarily inducing gene expression related to fatty acid oxidation in the liver of SHR/cp rats.

### TNK administration activates SIRT1 and AMPK signaling

**Fig 6:**
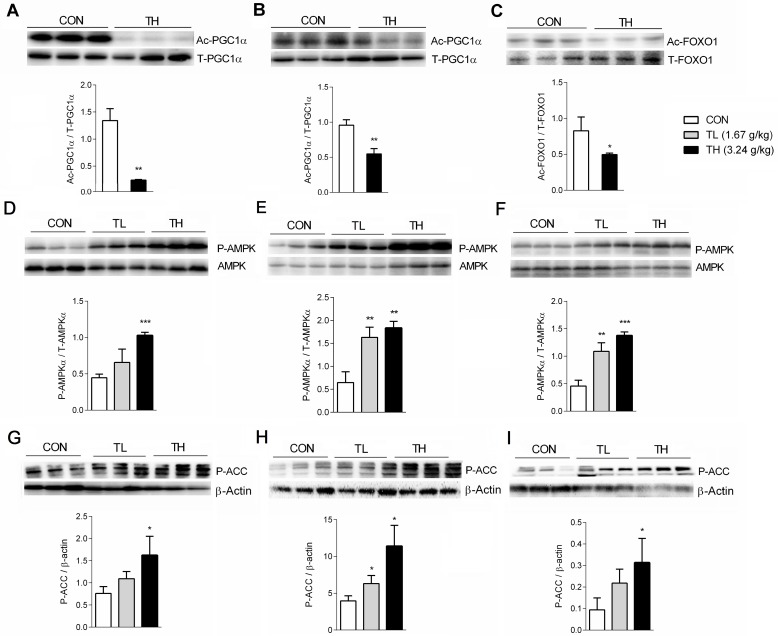
TNK promotes the deacetylation of PGC1α and FOXO1 and activates AMPK signaling. Acetylation levels of PGC1α in the skeletal muscle (A) and WAT (B) and FOXO1 acetylation level in the skeletal muscle (C) of SHR/cp rats treated with 3.24 g/kg TNK for 7 weeks are shown. Phosphorylation levels of AMPKα in the liver (D), skeletal muscle (E) and WAT (F) of SHR/cp rats treated with TNK for 7 weeks are shown. Phosphorylation levels of ACC in the liver (G), skeletal muscle (H) and WAT (I) of SHR/cp rats treated with TNK for 7 weeks relative to β-actin. Data are shown as the mean ± SD. *P < 0.05, **P < 0.01, ***P < 0.001 vs. CON. TNK, Tang-Nai-Kang; SHR/cp, SHR.Cg-*Lepr* ^cp^/NDmcr rat; WKY, Wistar Kyoto rat; CON, control group; TL, low-dose TNK group (1.67 g/kg); TH, high-dose TNK group (3.24 g/kg); WAT, white adipose tissue; PGC1α, peroxisome proliferator activated receptor-γ coactivator 1α; FOXO, forkhead transcription factor; AMPK, AMP-activated protein kinase; ACC, acetyl-CoA carboxylase.

TNK treatment significantly increased SIRT1 mRNA expression in the WAT, skeletal muscle and liver (Figs 3C, 4C and 5E). Furthermore, TNK induced gene expression related to fat oxidation at least partly through the transcriptional regulation of metabolic networks. Therefore, we examined the effect of TNK on the acetylation of SIRT1 transcriptional targets. As shown in Fig 6A and 6B, PGC1α expression in the skeletal muscle and WAT of TNK-treated SHR/cp rats was markedly deacetylated. Furthermore, TNK induced the deacetylation of FOXO1, another SIRT1 target associated with the regulation of metabolic homeostasis, in skeletal muscle (Fig 6C) [15,52]. These results demonstrate that TNK can activate SIRT1 signaling in SHR/cp rats.

We were also interested in investigating whether TNK could regulate AMPK expression and activity in SHR/cp rats. The phosphorylation levels of AMPKα and ACC were significantly elevated in the liver, muscle and WAT of SHR/cp rats treated with 1.67 g/kg and 3.24 g/kg TNK (Fig 6D, 6E, 6F, 6G, 6H and 6I), suggesting that TNK could activate AMPK signaling in SHR/cp rats.

## Discussion

In the present study, we confirmed that SHR/cp rats developed obvious MetS, characterized by obesity, glucose and lipid disorders, hypertension and fatty liver. The pre-diabetic state, characterized by moderately elevated fasting serum glucose and glucose intolerance, develops spontaneously in SHR/cp rats. When administered at a dose of 1.67 g/kg, TNK improved glucose homeostasis, dyslipidemia and insulin sensitivity in SHR/cp rats. These beneficial actions were even more pronounced at a dose of 3.24 g/kg, which also improved obesity, fatty liver and hypertension. Importantly the beneficial actions of TNK on glucose homeostasis and insulin sensitivity were likely to be indirect consequences of reduced serum lipid and fat mass, as they can only be recapitulated after long-term administration, in which serum lipid and body weight differences between groups are significant.

**Fig 7:**
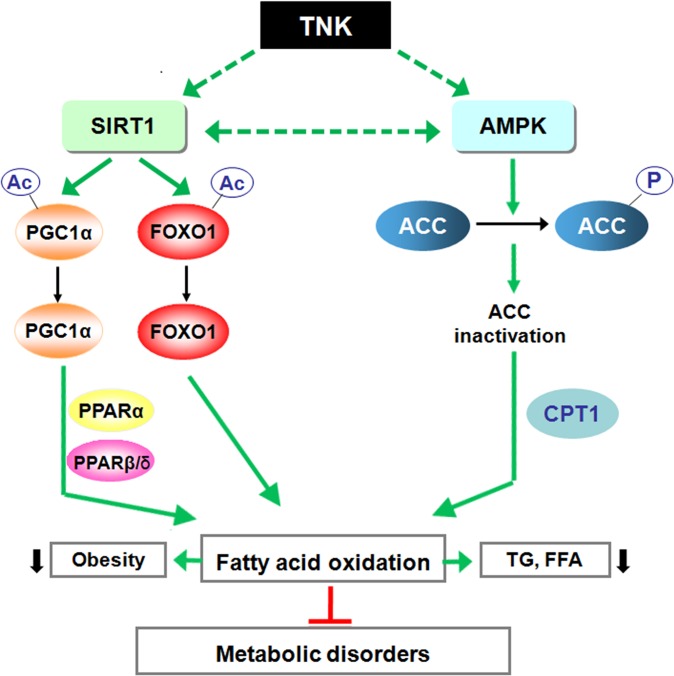
Schematic diagram of the potential mechanism of TNK protection against pre-diabetes and MetS in SHR/cp rats. TNK activates SIRT1 signaling in SHR/cp rats by up-regulating SIRT1 expression and increasing the deacetylation of PGC1α and FOXO1, two established targets of SIRT1. TNK activates AMPK signaling, as shown by the elevated AMPK and ACC phosphorylation levels in TNK-treated SHR/cp rats. In addition to the up-regulation of PPARα and PPARβ/δ, two nuclear receptors regulating fat oxidation, genes related to fat oxidation and energy expenditure are up-regulated, which in turn leads to improved hyperlipidemia, reduced adiposity and ameliorated pre-diabetes and MetS. TNK, Tang-Nai-Kang; SIRT1, sirtuin 1; PGC1α, peroxisome proliferator activated receptor-γ coactivator 1α; PPARα, PPARβ/δ, peroxisome proliferator activated receptor-α, -β/δ; PGC1α, peroxisome proliferator activated receptor-γ coactivator 1α; FOXO 1, forkhead transcription factor 1; AMPK, AMP-activated protein kinase; ACC, acetyl-CoA carboxylase; CPT, carnitine palmitoyltransferase; TG, total triglycerides; FFA, free fatty acids.

SIRT1 plays a vital role in increasing the rate of fat oxidation in response to low energy levels, and it has been speculated to be a metabolic modulator that switches the energy source from glucose to fatty acids in nutrient-deprived conditions such as fasting or calorie restriction (CR) [20,53]. In addition, AMPK has emerged as a key nutrient and energy status sensor. Upon activation, AMPK initiates catabolic pathways that restore ATP levels by promoting the use of mitochondrial substrates as an energy source to adapt to CR [54]. In the present study, the beneficial effects of TNK were associated with the activation of SIRT1 and AMPK signaling in SHR/cp rats, as TNK elevated SIRT1 mRNA expression, promoted the deacetylation of two well-established SIRT1 targets, PGC1α and FOXO1, and induced the phosphorylation of AMPK and ACC in different tissues. These effects seemed to activate pathways controlling fatty acid oxidation. Consistently, the mRNA expression of PPARα and PPARβ/δ, two major nuclear receptors that regulate fatty acid oxidation [55,56], were elevated by TNK treatment in skeletal muscle, liver and WAT in SHR/cp rats. Furthermore, oxidative PPAR target genes, such as CPT1α, LCAD, PDK4 and Acox1, were markedly up-regulated. These TNK-mediated gene expression changes were consistent with the improved hyperlipidemia, reduced fat mass and hepatic TG content in TNK-treated SHR/cp rats, which likely mimicked the metabolic alteration activated by CR and led to the improvement of pre-diabetes and metabolic disorders (Fig 7). Therefore, the switch in gene expression toward more oxidative capacity of skeletal muscle fiber types observed in TNK-treated SHR/cp rats most likely resulted from the deacetylation of PGC1α and FOXO1 following TNK treatment, as these transcriptional regulators are deacetylated by SIRT1 to enhance oxidative muscle function [52,57,58]. Because PGC1α is a transcriptional coactivator for PPARα- and PPARβ/δ-mediated fatty acid oxidation, activation of PGC1α by deacetylation likely synergized with the elevated PPAR expression to enhance oxidative metabolism [59,60]. Moreover, it is possible that the cooperative interplay between SIRT1 and AMPK signaling [29,30] also modulated the systemic metabolic effects induced by TNK in SHR/cp rats.

There are several limitations of this study. First, the main active fraction or compound(s) responsible for the beneficial effects of TNK are not known. In addition, we cannot confirm whether TNK has a direct effect on the activation of SIRT1 and AMPK or establish its interplay with the SIRT1-AMPK network. Finally, we do not know the direct effect of TNK on the induction of a metabolic adaption favoring energy expenditure and the utilization of fatty acids *in vivo*. We will further explore the active ingredients and functional characteristics of TNK in the future.

In summary, TNK improved the metabolic profile and lessened the pathological changes of pre-diabetes and MetS in SHR/cp rats, which were associated with the induction of a gene expression switch toward fatty acid oxidation. TNK induced this expression switch by inducing the deacetylation of SIRT1 targets and the activation of AMPK in SHR/cp rats. The current study suggests that TNK may prove to be a promising agent for treating certain diseases associated with energy surplus and obesity, such as pre-diabetes and MetS.

## Supporting Information

S1 Fig Typical chromatograms for the determination of RA in TNK. (A) Chromatogram of the reference standard of RA. (B) Chromatogram of the TNK extract. TNK, Tang-Nai-Kang; RA, rosmarinic acid.
(TIF)

S2 Fig Typical chromatograms for the determination of ginsenosides Rg1 and Re in TNK. (A) Chromatogram of the reference standards of Rg1 and Re. (B) Chromatogram of the TNK extract. TNK, Tang-Nai-Kang.
(TIF)

S3 Fig Typical chromatograms for the determination of ginsenosides Rb1 in TNK. (A) Chromatogram of the reference standards of Rb1. (B) Chromatogram of the TNK extract. TNK, Tang-Nai-Kang.
(TIF)

S1 Table Spectrophotometry analysis of TNK. TNK, Tang-Nai-Kang; TWE, TNK water extract; TEE, TNK ethanol extract; TP, total polysaccharides; TF, total flavonoids; TS, triterpenoid saponin; RSD, relative standard deviation; Abs, absorbance.
(PDF)

S2 Table Rat primers used for PCR analysis. Acox1, acetyl-CoA carboxylase 1; AdipoR2, adiponectin receptor 2; CEBPα, CCAAT/enhancer-binding protein α; CPT1α, carnitine palmitoyltransferase 1α; CoxIV, cytochrome c oxidase subunit IV; ERRα, estrogen related receptor α; FABP4, fatty acid-binding protein 4; FOXO1, forkhead transcription factor 1; G6Pase, glucose-6-phosphatase; GCK, glucokinase; HK2, hexokinase 2; HSL, hormone-sensitive lipase; LCAD, long-chain-acyl-CoA dehydrogenase; MHCI, MHCIIa, MHCIIb, myosin heavy chains, type I, IIa, IIb; PDK4, pyruvate dehydrogenase kinase 4; PEPCK, phosphoenolpyruvate carboxykinase; PFKFB3, 6-phosphofructo-2-kinase/fructose-2, 6-biphosphatase 3; PGC1α, peroxisome proliferator-activated receptor γ coactivator 1α; PPARα, PPARβ/δ, PPARγ, peroxisome proliferator activated receptor-α, -β/δ, -γ; SIRT1, sirtuin 1; TFAM, mitochondrial transcription factor; TnI(slow), slow twitch skeletal muscle troponin I. Actin was used as reference gene.
(PDF)

S1 Text Spectrophotometry analysis method. (PDF)

S2 Text High-performance liquid chromatography (HPLC) analysis method. (PDF)

## References

[1] Chiolero A, Paccaud F. Prediabetes and the risk of diabetes. Lancet. 2012;380: 1225; author reply 1226. 10.1016/S0140-6736(12)61704-8 23040851

[2] Deedwania P, Ahmed A. Prediabetes and the risk of diabetes. Lancet. 2012;380: 1225; author reply 1226. 10.1016/S0140-6736(12)61704-8 23040849

[3] Tabak AG, Herder C, Rathmann W, Brunner EJ, Kivimaki M. Prediabetes: a high-risk state for diabetes development. Lancet. 2012;379: 2279–2290. 10.1016/S0140-6736(12)60283-9 22683128 PMC3891203

[4] Grundy SM. Pre-diabetes, metabolic syndrome, and cardiovascular risk. J Am Coll Cardiol. 2012;59: 635–643. 10.1016/j.jacc.2011.08.080 22322078

[5] Stern MP, Williams K, Gonzalez-Villalpando C, Hunt KJ, Haffner SM. Does the metabolic syndrome improve identification of individuals at risk of type 2 diabetes and/or cardiovascular disease. Diabetes Care. 2004;27: 2676–2681. 15505004 10.2337/diacare.27.11.2676

[6] Lorenzo C, Williams K, Hunt KJ, Haffner SM. The National Cholesterol Education Program—Adult Treatment Panel III, International Diabetes Federation, and World Health Organization definitions of the metabolic syndrome as predictors of incident cardiovascular disease and diabetes. Diabetes Care. 2007;30: 8–13. 17192325 10.2337/dc06-1414

[7] Florez H, Temprosa MG, Orchard TJ, Mather KJ, Marcovina SM, Barrett-Connor E, et al Metabolic syndrome components and their response to lifestyle and metformin interventions are associated with differences in diabetes risk in persons with impaired glucose tolerance. Diabetes Obes Metab. 2014;16: 326–333. 10.1111/dom.12220 24118860 PMC3943638

[8] Knowler WC, Barrett-Connor E, Fowler SE, Hamman RF, Lachin JM, Walker EA, et al Reduction in the incidence of type 2 diabetes with lifestyle intervention or metformin. N Engl J Med. 2002;346: 393–403. 11832527 10.1056/NEJMoa012512 PMC1370926

[9] Knowler WC, Hamman RF, Edelstein SL, Barrett-Connor E, Ehrmann DA, Walker EA, et al Prevention of type 2 diabetes with troglitazone in the Diabetes Prevention Program. Diabetes. 2005;54: 1150–1156. 15793255 10.2337/diabetes.54.4.1150 PMC1351025

[10] Moore EM, Mander AG, Ames D, Kotowicz MA, Carne RP, Brodaty H, et al Increased risk of cognitive impairment in patients with diabetes is associated with metformin. Diabetes Care. 2013;36: 2981–2987. 10.2337/dc13-0229 24009301 PMC3781568

[11] Desvergne B, Michalik L, Wahli W. Transcriptional regulation of metabolism. Physiol Rev. 2006;86: 465–514. 16601267 10.1152/physrev.00025.2005

[12] Feige JN, Auwerx J. Transcriptional coregulators in the control of energy homeostasis. Trends Cell Biol. 2007;17: 292–301. 17475497 10.1016/j.tcb.2007.04.001

[13] Guarente L. Sirtuins as potential targets for metabolic syndrome. Nature. 2006;444: 868–874. 17167475 10.1038/nature05486

[14] Chang HC, Guarente L. SIRT1 and other sirtuins in metabolism. Trends Endocrinol Metab. 2014;25: 138–145. 10.1016/j.tem.2013.12.001 24388149 PMC3943707

[15] Feige JN, Auwerx J. Transcriptional targets of sirtuins in the coordination of mammalian physiology. Curr Opin Cell Biol. 2008;20: 303–309. 10.1016/j.ceb.2008.03.012 18468877 PMC2447870

[16] Wang Y, Xu C, Liang Y, Vanhoutte PM. SIRT1 in metabolic syndrome: where to target matters. Pharmacol Ther. 2012;136: 305–318. 10.1016/j.pharmthera.2012.08.009 22939883

[17] Gerhart-Hines Z, Rodgers JT, Bare O, Lerin C, Kim SH, Mostoslavsky R, et al Metabolic control of muscle mitochondrial function and fatty acid oxidation through SIRT1/PGC-1alpha. EMBO J. 2007;26: 1913–1923. 17347648 10.1038/sj.emboj.7601633 PMC1847661

[18] Lagouge M, Argmann C, Gerhart-Hines Z, Meziane H, Lerin C, Daussin F, et al Resveratrol improves mitochondrial function and protects against metabolic disease by activating SIRT1 and PGC-1alpha. Cell. 2006;127: 1109–1122. 17112576 10.1016/j.cell.2006.11.013

[19] Liang F, Kume S, Koya D. SIRT1 and insulin resistance. Nat Rev Endocrinol. 2009;5: 367–373. 10.1038/nrendo.2009.101 19455179

[20] Rodgers JT, Lerin C, Haas W, Gygi SP, Spiegelman BM, Puigserver P. Nutrient control of glucose homeostasis through a complex of PGC-1alpha and SIRT1. Nature. 2005;434: 113–118. 15744310 10.1038/nature03354

[21] Hardie DG, Ashford ML. AMPK: regulating energy balance at the cellular and whole body levels. Physiology (Bethesda). 2014;29: 99–107. 10.1152/physiol.00050.2013 24583766 PMC3949207

[22] Hardie DG, Ross FA, Hawley SA. AMPK: a nutrient and energy sensor that maintains energy homeostasis. Nat Rev Mol Cell Biol. 2012;13: 251–262. 10.1038/nrm3311 22436748 PMC5726489

[23] Zhang BB, Zhou G, Li C. AMPK: an emerging drug target for diabetes and the metabolic syndrome. Cell Metab. 2009;9: 407–416. 10.1016/j.cmet.2009.03.012 19416711

[24] Merrill GF, Kurth EJ, Hardie DG, Winder WW. AICA riboside increases AMP-activated protein kinase, fatty acid oxidation, and glucose uptake in rat muscle. Am J Physiol. 1997;273: E1107–1112. 9435525 10.1152/ajpendo.1997.273.6.E1107

[25] Atkinson LL, Fischer MA, Lopaschuk GD. Leptin activates cardiac fatty acid oxidation independent of changes in the AMP-activated protein kinase-acetyl-CoA carboxylase-malonyl-CoA axis. J Biol Chem. 2002;277: 29424–29430. 12058043 10.1074/jbc.M203813200

[26] Bronner M, Hertz R, Bar-Tana J. Kinase-independent transcriptional co-activation of peroxisome proliferator-activated receptor alpha by AMP-activated protein kinase. Biochem J. 2004;384: 295–305. 15312046 10.1042/BJ20040955 PMC1134113

[27] Jager S, Handschin C, St-Pierre J, Spiegelman BM. AMP-activated protein kinase (AMPK) action in skeletal muscle via direct phosphorylation of PGC-1alpha. Proc Natl Acad Sci U S A. 2007;104: 12017–12022. 17609368 10.1073/pnas.0705070104 PMC1924552

[28] Barroso E, Eyre E, Palomer X, Vazquez-Carrera M. The peroxisome proliferator-activated receptor beta/delta (PPARbeta/delta) agonist GW501516 prevents TNF-alpha-induced NF-kappaB activation in human HaCaT cells by reducing p65 acetylation through AMPK and SIRT1. Biochem Pharmacol. 2011;81: 534–543. 10.1016/j.bcp.2010.12.004 21146504

[29] Canto C, Gerhart-Hines Z, Feige JN, Lagouge M, Noriega L, Milne JC, et al AMPK regulates energy expenditure by modulating NAD^+^ metabolism and SIRT1 activity. Nature. 2009;458: 1056–1060. 10.1038/nature07813 19262508 PMC3616311

[30] Canto C, Auwerx J. PGC-1alpha, SIRT1 and AMPK, an energy sensing network that controls energy expenditure. Curr Opin Lipidol. 2009;20: 98–105. 10.1097/MOL.0b013e328328d0a4 19276888 PMC3627054

[31] Baur JA, Pearson KJ, Price NL, Jamieson HA, Lerin C, Kalra A, et al Resveratrol improves health and survival of mice on a high-calorie diet. Nature. 2006;444: 337–342. 17086191 10.1038/nature05354 PMC4990206

[32] Feige JN, Lagouge M, Canto C, Strehle A, Houten SM, Milne JC, et al Specific SIRT1 activation mimics low energy levels and protects against diet-induced metabolic disorders by enhancing fat oxidation. Cell Metab. 2008;8: 347–358. 10.1016/j.cmet.2008.08.017 19046567

[33] Wang HJ, Chiang BH. Anti-diabetic effect of a traditional Chinese medicine formula. Food Funct. 2012;3: 1161–1169. 10.1039/c2fo30139c 22899105

[34] Grant SJ, Chang DH, Liu J, Wong V, Kiat H, Bensoussan A. Chinese herbal medicine for impaired glucose tolerance: a randomized placebo controlled trial. BMC Complement Altern Med. 2013;13: 104 10.1186/1472-6882-13-104 23672597 PMC3659077

[35] Nan XJ, Qin ZD, Du J, Wen J. Regulation effects of TZQ-F on adipocyte differentiation and insulin action. J Ethnopharmacol. 2013;150: 692–699. 10.1016/j.jep.2013.09.038 24095827

[36] Li JB, Xu LJ, Dong H, Huang ZY, Zhao Y, Chen G, et al Effects of Chinese Fructus Mume formula and its separated prescription extract on insulin resistance in type 2 diabetic rats. J Huazhong Univ Sci Technolog Med Sci. 2013;33: 877–885. 10.1007/s11596-013-1215-7 24337852

[37] Hwang SM, Kim JS, Lee YJ, Yoon JJ, Lee SM, Kang DG, et al Anti-diabetic atherosclerosis effect of *Prunella vulgaris* in db/db mice with type 2 diabetes. Am J Chin Med. 2012;40: 937–951. 10.1142/S0192415X12500693 22928826

[38] Wang L, Cheng D, Wang H, Di L, Zhou X, Xu T, et al The hepatoprotective and antifibrotic effects of *Saururus chinensis* against carbon tetrachloride induced hepatic fibrosis in rats. J Ethnopharmacol. 2009;126: 487–491. 10.1016/j.jep.2009.09.009 19761824

[39] Gao D, Li Q, Li Y, Liu Z, Fan Y, Liu Z, et al Antidiabetic and antioxidant effects of oleanolic acid from *Ligustrum lucidum* Ait in alloxan-induced diabetic rats. Phytother Res. 2009;23: 1257–1262. 10.1002/ptr.2603 19274687

[40] Guo X, Yoshitomi H, Gao M, Qin L, Duan Y, Sun W, et al Guava leaf extracts promote glucose metabolism in SHRSP.Z-Leprfa/Izm rats by improving insulin resistance in skeletal muscle. BMC Complement Altern Med. 2013;13: 52 10.1186/1472-6882-13-52 23452929 PMC3599057

[41] Yuan HD, Kim JT, Kim SH, Chung SH. Ginseng and diabetes: the evidences from *in vitro*, animal and human studies. J Ginseng Res. 2012;36: 27–39. 10.5142/jgr.2012.36.1.27 23717101 PMC3659569

[42] Shishtar E, Sievenpiper JL, Djedovic V, Cozma AI, Ha V, Jayalath VH, et al The effect of ginseng (the genus *panax*) on glycemic control: a systematic review and meta-analysis of randomized controlled clinical trials. PLoS One. 2014;9: e107391 10.1371/journal.pone.0107391 25265315 PMC4180277

[43] Liu TH. The improvement effect of Tang-Nai-Kang on glucose intolerance and insulin resistance. China Science and Technology Achievements. 2011;12: 79. Chinese.

[44] Guo XY, Duan Y, Li JE, Yang LX, Huang LS, Wang ZC, et al Antidiabetic effects of Tangnaikang on obese Zucker rats and the mechanism. Zhong Xi Yi Jie He Xue Bao. 2010;8: 535–540. 20550875 10.3736/jcim20100605

[45] Kawai K, Sakairi T, Harada S, Shinozuka J, Ide M, Sato H, et al Diet modification and its influence on metabolic and related pathological alterations in the SHR/NDmcr-cp rat, an animal model of the metabolic syndrome. Exp Toxicol Pathol. 2012;64: 333–338. 10.1016/j.etp.2010.09.006 20965707

[46] Nagase M, Yoshida S, Shibata S, Nagase T, Gotoda T, Ando K, et al Enhanced aldosterone signaling in the early nephropathy of rats with metabolic syndrome: possible contribution of fat-derived factors. J Am Soc Nephrol. 2006;17: 3438–3446. 17082236 10.1681/ASN.2006080944

[47] Liu TH. The preparation method of Tang-Nai-Kang granule. 2004. PR China Patent. CN02153751.8. Chinese.

[48] Matthews DR, Hosker JP, Rudenski AS, Naylor BA, Treacher DF, Turner RC. Homeostasis model assessment: insulin resistance and beta-cell function from fasting plasma glucose and insulin concentrations in man. Diabetologia. 1985;28: 412–419. 3899825 10.1007/BF00280883

[49] Lowry OH, Rosebrough NJ, Farr AL, Randall RJ. Protein measurement with the Folin phenol reagent. J Biol Chem. 1951;193: 265–275. 14907713

[50] Smith DL, Johnson M, Nagy T. Precision and accuracy of bioimpedance spectroscopy for determination of in vivo body composition in rats. Int J Body Compos Res. 2009;7: 21–26. 19668348 PMC2722071

[51] Li L, Gao L, Liu S, Liu Q, Sun S, Huan Y, et al Bis(alpha-furancarboxylato)oxovanadium(IV) exerts durable antidiabetic effects and suppresses matrix metalloproteinase-2 activity in spontaneous type 2 diabetic KKAy mice. Biol Trace Elem Res. 2013;153: 329–339. 10.1007/s12011-013-9689-5 23649370

[52] Gross DN, van den Heuvel AP, Birnbaum MJ. The role of FoxO in the regulation of metabolism. Oncogene. 2008;27: 2320–2336. 10.1038/onc.2008.25 18391974

[53] Michan S, Sinclair D. Sirtuins in mammals: insights into their biological function. Biochem J. 2007;404: 1–13. 17447894 10.1042/BJ20070140 PMC2753453

[54] Canto C, Auwerx J. Calorie restriction: is AMPK a key sensor and effector. Physiology (Bethesda). 2011;26: 214–224. 10.1152/physiol.00010.2011 21841070 PMC3627048

[55] Menendez-Gutierrez MP, Roszer T, Ricote M. Biology and therapeutic applications of peroxisome proliferator- activated receptors. Curr Top Med Chem. 2012;12: 548–584. 22242855 10.2174/156802612799436669

[56] Feige JN, Gelman L, Michalik L, Desvergne B, Wahli W. From molecular action to physiological outputs: peroxisome proliferator-activated receptors are nuclear receptors at the crossroads of key cellular functions. Prog Lipid Res. 2006;45: 120–159. 16476485 10.1016/j.plipres.2005.12.002

[57] Lin J, Wu H, Tarr PT, Zhang CY, Wu Z, Boss O, et al Transcriptional co-activator PGC-1 alpha drives the formation of slow-twitch muscle fibres. Nature. 2002;418: 797–801. 12181572 10.1038/nature00904

[58] Wang L, Jia Y, Rogers H, Suzuki N, Gassmann M, Wang Q, et al Erythropoietin contributes to slow oxidative muscle fiber specification via PGC-1alpha and AMPK activation. Int J Biochem Cell Biol. 2013;45: 1155–1164. 10.1016/j.biocel.2013.03.007 23523698 PMC3684177

[59] Vega RB, Huss JM, Kelly DP. The coactivator PGC-1 cooperates with peroxisome proliferator-activated receptor alpha in transcriptional control of nuclear genes encoding mitochondrial fatty acid oxidation enzymes. Mol Cell Biol. 2000;20: 1868–1876. 10669761 10.1128/mcb.20.5.1868-1876.2000 PMC85369

[60] Wang YX, Lee CH, Tiep S, Yu RT, Ham J, Kang H, et al Peroxisome-proliferator-activated receptor delta activates fat metabolism to prevent obesity. Cell. 2003;113: 159–170. 12705865 10.1016/s0092-8674(03)00269-1

