# Supplementary figures and images for: Tang-Nai-Kang Alleviates Pre-diabetes and Metabolic Disorders and Induces a Gene Expression Switch toward Fatty Acid Oxidation in SHR.Cg-Leprcp/NDmcr Rats
Source: PLoS One. 2015 Apr 13;10(4):e0122024. doi: 10.1371/journal.pone.0122024 (PMC4395456; doi:10.1371/journal.pone.0122024)

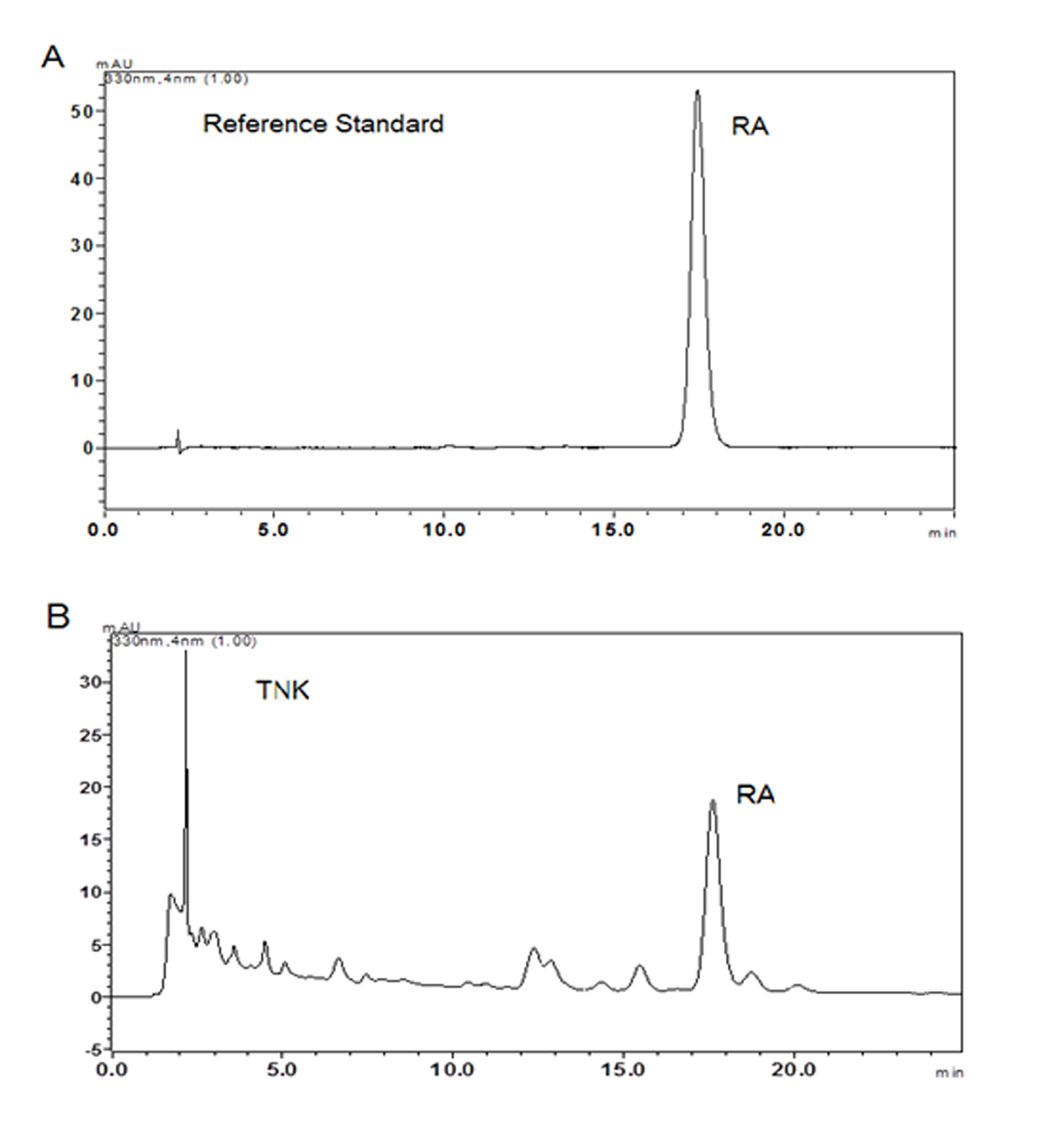

Supplement: S1 Fig — (A) Chromatogram of the reference standard of RA. (B) Chromatogram of the TNK extract. TNK, Tang-Nai-Kang; RA, rosmarinic acid. (TIF) [file pone.0122024.s001.tif]

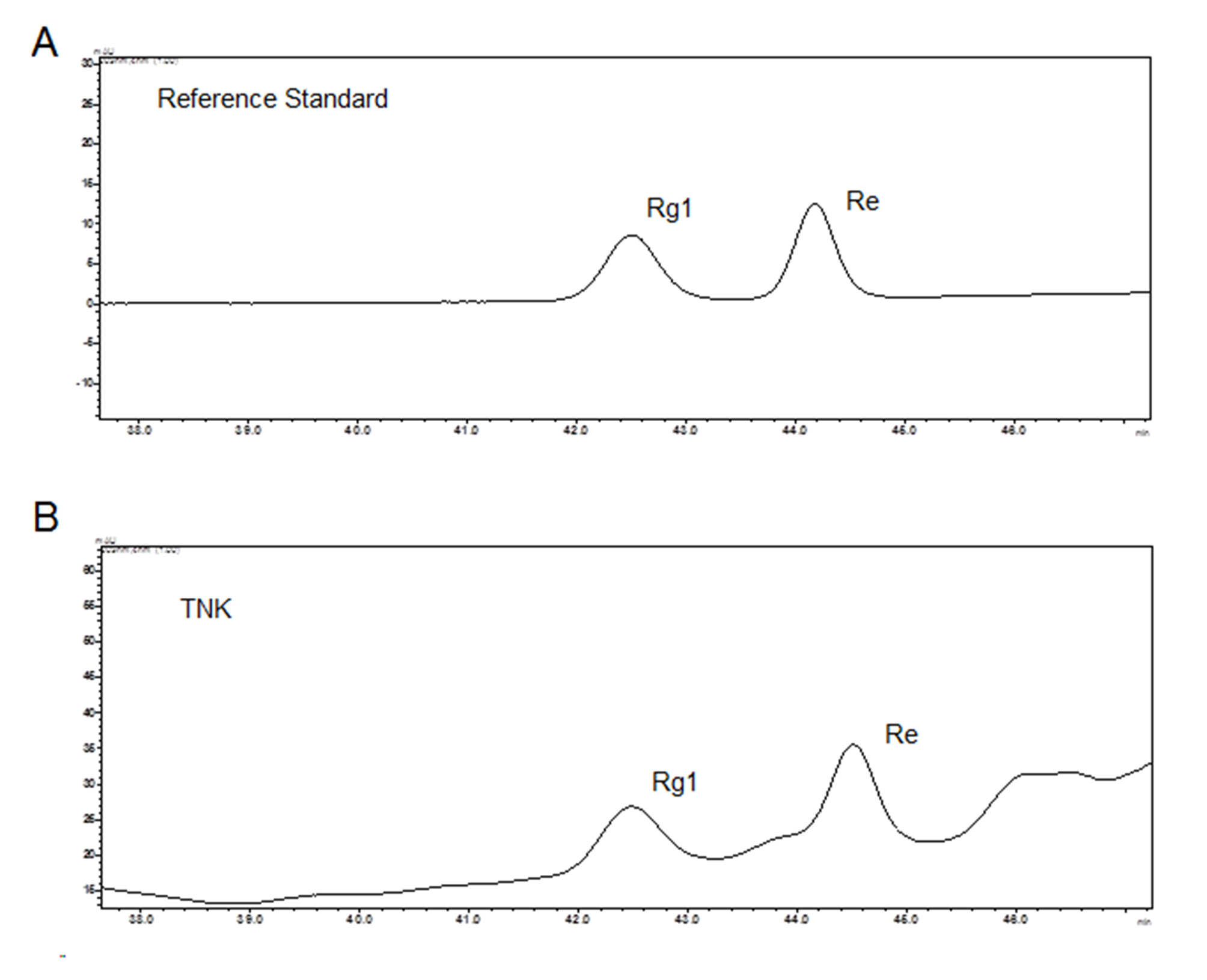

Supplement: S2 Fig — (A) Chromatogram of the reference standards of Rg1 and Re. (B) Chromatogram of the TNK extract. TNK, Tang-Nai-Kang. (TIF) [file pone.0122024.s002.tif]

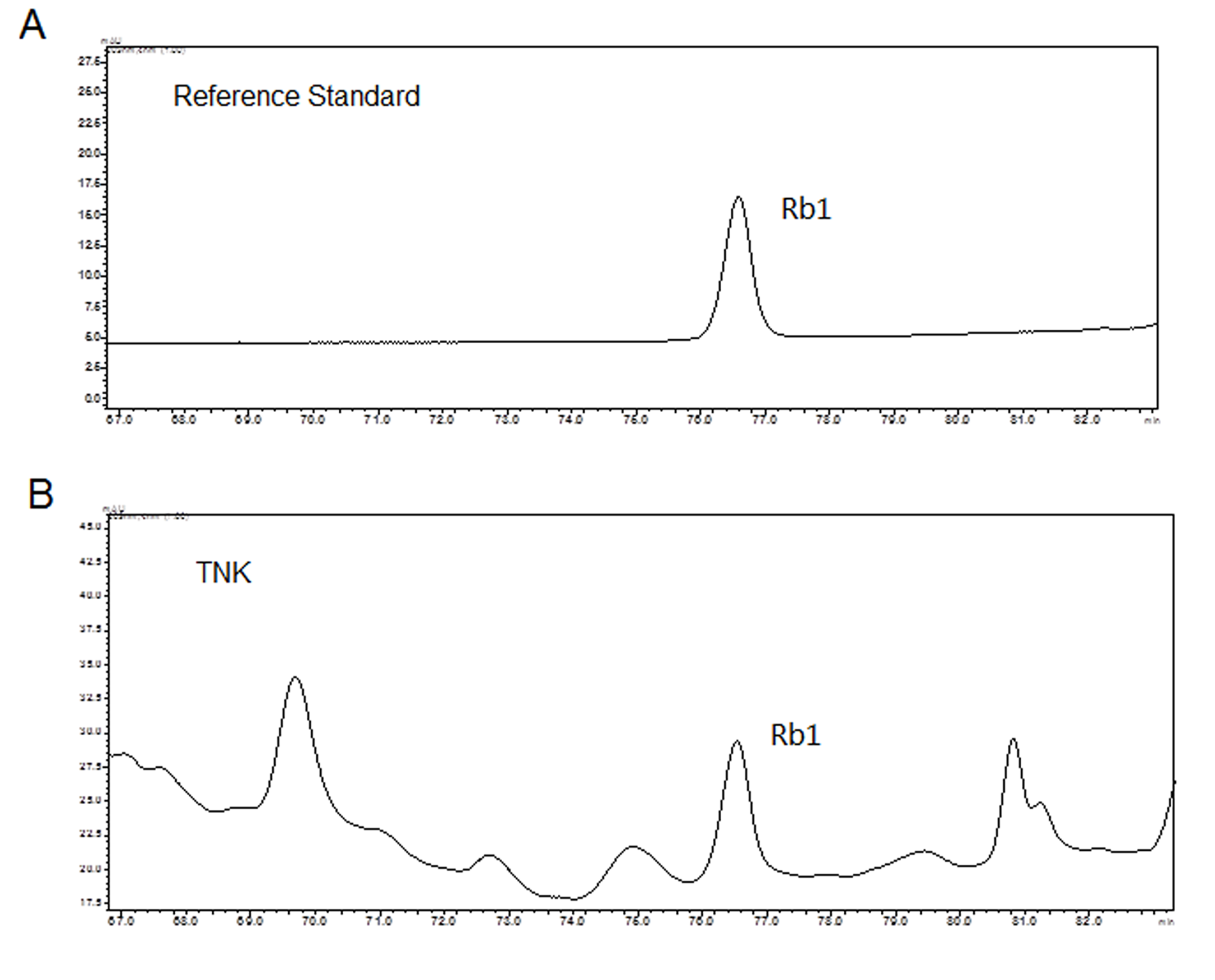

Supplement: S3 Fig — (A) Chromatogram of the reference standards of Rb1. (B) Chromatogram of the TNK extract. TNK, Tang-Nai-Kang. (TIF) [file pone.0122024.s003.tif]
