# Supplementary material for: Tang-Nai-Kang Alleviates Pre-diabetes and Metabolic Disorders and Induces a Gene Expression Switch toward Fatty Acid Oxidation in SHR.Cg-Leprcp/NDmcr Rats
Source: PLoS One. 2015 Apr 13;10(4):e0122024. doi: 10.1371/journal.pone.0122024 (PMC4395456; doi:10.1371/journal.pone.0122024)
Supplement: S1 Table — TNK, Tang-Nai-Kang; TWE, TNK water extract; TEE, TNK ethanol extract; TP, total polysaccharides; TF, total flavonoids; TS, triterpenoid saponin; RSD, relative standard deviation; Abs, absorbance. (PDF) [file pone.0122024.s004.pdf]

S1 Table. Spectrophotometry analysis of TNK.

Extract	Product	Abs	Yield (% w/w)	Mean yield (% w/w)	RSD/%
TWE	TP	0.5868	5.75	5.83	1.24
		0.6011	5.87		
		0.6023	5.88		
TEE	TF	0.2415	4.20	4.23	0.72
		0.2461	4.26		
		0.2446	4.24		
TEE	TS	0.2965	3.63	3.67	1.10
		0.3059	3.71		
		0.3000	3.66		

TNK, Tang-Nai-Kang; TWE, TNK water extract; TEE, TNK ethanol extract; TP, total polysaccharides; TF, total flavonoids; TS, triterpenoid saponin; RSD, relative standard deviation; Abs, absorbance.
