# Supplementary material for: Tang-Nai-Kang Alleviates Pre-diabetes and Metabolic Disorders and Induces a Gene Expression Switch toward Fatty Acid Oxidation in SHR.Cg-Leprcp/NDmcr Rats
Source: PLoS One. 2015 Apr 13;10(4):e0122024. doi: 10.1371/journal.pone.0122024 (PMC4395456; doi:10.1371/journal.pone.0122024)
Supplement: S2 Table — Acox1, acetyl-CoA carboxylase 1; AdipoR2, adiponectin receptor 2; CEBPα, CCAAT/enhancer-binding protein α; CPT1α, carnitine palmitoyltransferase 1α; CoxIV, cytochrome c oxidase subunit IV; ERRα, estrogen related receptor α; FABP4, fatty acid-binding protein 4; FOXO1, forkhead transcription factor 1; G6Pase, glucose-6-phosphatase; GCK, glucokinase; HK2, hexokinase 2; HSL, hormone-sensitive lipase; LCAD, long-chain-acyl-CoA dehydrogenase; MHCI, MHCIIa, MHCIIb, myosin heavy chains, type I, IIa, IIb; PDK4, pyruvate dehydrogenase kinase 4; PEPCK, phosphoenolpyruvate carboxykinase; PFKFB3, 6-phosphofructo-2-kinase/fructose-2, 6-biphosphatase 3; PGC1α, peroxisome proliferator-activated receptor γ coactivator 1α; PPARα, PPARβ/δ, PPARγ, peroxisome proliferator activated receptor-α, -β/δ, -γ; SIRT1, sirtuin 1; TFAM, mitochondrial transcription factor; TnI(slow), slow twitch skeletal muscle troponin I. Actin was used as reference gene. (PDF) [file pone.0122024.s005.pdf]

S2 Table. Rat primers used for PCR analysis.

No.		Forward primer sequence	Reverse primer sequence
1	SIRT1	TGAAGCTGTTCGTGGAGATATTTTT	CATGATGGCAAGTGGCTCAT
2	PPARγ	GAGATCCTCCTGTTGACCCAG	CCACAGAGCTGATTCCGAAGT
3	CEBPα	TACCGAGTAGGGGGAGCAAA	CCTCTAAGGACAGGGACGGA
4	FABP4	AGAACTCACTGGGACCTGGA	TCTCGGACAGCAATCAGCTC
5	HSL	AGTGAAAAACCCGCGGACC	TTCATCCTTCTGCCCCCTAC
6	PGC1α	TGGAGTGACATAGAGTGTGCTG	TATGTTCGCGGGCTCATTGT
7	PPARα	GTCCTCTGGTTGTCCCCTTG	GTCAGTTCACAGGGAAGGCA
8	PPARβ/δ	GTATCCGCAAGCCCTTCAGT	GATGGCGGCAATGAAAAGGG
9	HK2	CTGGTGAGCCATCGTGGTTA	AAGCAGGCGATCATATGCGA
10	PFKFB3	CGCAATAGTGTCACCCCACT	TCCCTAGCAAAGGTTGTCCG
11	FOXO1	CAGGCCGGAGTTTAACCAGT	CTCGCTCTCTTCTAGCAGGC
12	ERRα	GGTGGCCGACAGAAGTACAA	GGCGTACAGCTTCTCAGGTT
13	TFAM	TTCCAGGGGGCTAAGGATGA	CACACTGCGACGGATGAGAT
14	LCAD	CCCTGGTTTCAGCCTCCATT	TCACTCCCAGACCTTTTGGC
15	MCAD	AGCCTTCACCGGATTCATCG	AGCCCCCATTGCAATCTTGA
16	PDK4	TATCGACCCCAACTGCGATG	TGGATTGGTTGGCCTGGAAA
17	GCK	CCTGGGAGGAACCAACTTCA	TCTTGTGCTTCATCATCTCGGC
18	PEPCK	TGCCCAAGATCTTCCACGTC	TCAAGTTCAGGGCGTCTTCC
19	G6Pase	GGACCTCCTGTGGACTTTGG	AAACGGAATGGGAGCGACTT
20	Acox1	CTCACTCGAAGCCAGCGTTA	TTGAGGCCAACAGGTTCCAC

21	AdipoR2	GGGTCAGAGCAGGAGTGTTC	AGAGGGCAGCTCCTGTGATA
22	Tnl(Slow)	GGTGGCAGAGCGAGAAGAG	TTTTCCGCTGCTCAAAGTGC
23	MHCIIa	CCGCGAGGTTCACACTAAAG	TGCCTCTCTTCGGTCATTCT
24	MHCIIb	AAGAGCCGAGAGGTTCACAC	TGTCACCTTTCAACAGAAGGAA
25	MHCI	ACCTGATGGTGGATGTGGAG	CTTCTGCTTCCACTCAACCA
26	CoxIV	CTCGAGCGAGATGGCTTCAA	GTAGGGACACCACCTCCAGA
27	Actin	CGTCTTCCCCTCCATCGT	GGAGTCCTTCTGACCCATACC

Acox1, acetyl-CoA carboxylase 1; AdipoR2, adiponectin receptor 2; CEBPα, CCAAT/enhancer-binding protein α; CPT1α, carnitine palmitoyltransferase 1α; CoxIV, cytochrome c oxidase subunit IV; ERRα, estrogen related receptor α; FABP4, fatty acid-binding protein 4; FOXO1, forkhead transcription factor 1; G6Pase, glucose-6-phosphatase; GCK, glucokinase; HK2, hexokinase 2; HSL, hormone-sensitive lipase; LCAD, long-chain-acyl-CoA dehydrogenase; MHCI, MHCIIa, MHCIIb, myosin heavy chains, type I, IIa, IIb; PDK4, pyruvate dehydrogenase kinase 4; PEPCK, phosphoenolpyruvate carboxykinase; PFKFB3, 6-phosphofructo-2-kinase/fructose-2, 6-biphosphatase 3; PGC1α, peroxisome proliferator-activated receptor γ coactivator 1α; PPARα, PPARβ/δ, PPARγ, peroxisome proliferator activated receptor-α, -β/δ, -γ; SIRT1, sirtuin 1; TFAM, mitochondrial transcription factor; TnI(slow), slow twitch skeletal muscle troponin I. Actin was used as reference gene.
