# Supplementary material for: Tang-Nai-Kang Alleviates Pre-diabetes and Metabolic Disorders and Induces a Gene Expression Switch toward Fatty Acid Oxidation in SHR.Cg-Leprcp/NDmcr Rats
Source: PLoS One. 2015 Apr 13;10(4):e0122024. doi: 10.1371/journal.pone.0122024 (PMC4395456; doi:10.1371/journal.pone.0122024)
Supplement: S1 Text — (PDF) [file pone.0122024.s006.pdf]

# S1 Text. Spectrophotometry analysis method.

## Determination of total polysaccharides (TP) in the water extract of TNK (TWE)

TWE (1.0 g) was precipitated using 95% (v/v) ethanol and then separated by centrifugation (3000 × g for 5 min). The precipitate was dissolved in deionized water, and the TP content was measured using the phenol-sulfuric colorimetric method at 490 nm, with glucose as a standard [1]. The TP yield (% w/w) was calculated using the following equation: TP yield (% w/w) = total polysaccharides weight/water extract weight × 100.

## Determination of total flavonoids (TF) in the ethanol extract of TNK (TEE)

Extraction of TF from TEE (2.0 g) was performed as described previously, and the TF content was measured using the aluminum chloride method at 410 nm, with rutin as a standard [2]. The TF yield (% w/w) was calculated using the following equation: TF yield (% w/w) = total flavonoids weight/ethanol extract weight × 100.

## Determination of triterpenoid saponin (TS) in the TEE

Using ursolic acid as a standard, TS in the TEE was determined using the spectrophotometry method at 550 nm, with 5% vanillin-glacial acetic acid solution and perchloric acid as color-developing agents [3]. The TS yield (% w/w) was calculated using the following equation: TS yield (% w/w) = total saponin weight/ethanol extract weight × 100.

# References

1. Koh GY, Chou G, Liu Z (2009) Purification of a water extract of Chinese sweet tea plant (Rubus suavissimus S. Lee) by alcohol precipitation. J Agric Food Chem 57: 5000-5006.
2. Wang Q, Jiang C, Fang S, Wang J, Ji Y, et al. (2013) Antihyperglycemic, antihyperlipidemic and antioxidant effects of ethanol and aqueous extracts of Cyclocarya paliurus leaves in type 2 diabetic rats. J Ethnopharmacol 150: 1119-1127.
3. Gao XH, Xu XX, Pan R, Li Y, Luo YB, et al. (2009) Saponin fraction from Astragalus membranaceus roots protects mice against polymicrobial sepsis induced by cecal ligation and puncture by inhibiting inflammation and upregulating protein C pathway. J Nat Med 63: 421-429.
