# Supplementary material for: Tang-Nai-Kang Alleviates Pre-diabetes and Metabolic Disorders and Induces a Gene Expression Switch toward Fatty Acid Oxidation in SHR.Cg-Leprcp/NDmcr Rats
Source: PLoS One. 2015 Apr 13;10(4):e0122024. doi: 10.1371/journal.pone.0122024 (PMC4395456; doi:10.1371/journal.pone.0122024)
Supplement: S2 Text — (PDF) [file pone.0122024.s007.pdf]

S2 Text. High-performance liquid chromatography (HPLC) analysis method.

Determination of rosmarinic acid in TNK

TNK was dissolved in diluted ethanol (10 mg/mL) and sonicated (power 90 W, frequency 59 Hz) for 30 min. The solution was filtered, and the subsequent filtrate was then collected for analysis. The rosmarinic acid content in TNK was determined on a Shimadzu Essentia LC-15C HPLC system (Shimadzu, Kyoto, Japan) with a Thermo Syncronis C18 column (150 mm × 4.6 mm, 5 μm, Thermo Scientific, New Jersey, USA). The mobile phase was a methanol/0.1% trifluoroacetic acid solution (42:58, v/v), and the flow rate was 1.0 mL/min. The analysis was performed at 30 ℃, and the absorbance at 330 nm was detected. The rosmarinic acid content was 0.55% when compared with the standards. Rosmarinic acid (RA; purity > 98%) was purchased from the National Institutes for Food and Drug Control (Beijing, China).

Determination of the ginsenosides Rg1, Re and Rb1 in TNK

TNK (1.0 g) was refluxed with sufficient chloroform at 80 ℃ for 3 hrs and then volatilized in a ventilated area [1]. The degreased sample was placed into a conical flask with 50 mL of water-saturated butanol. The conical flask was sealed and placed at room temperature for 12 hrs. Next, the sample was sonicated (power 250 W, frequency 50 Hz) for 30 min and filtered. The subsequent filtrate was then evaporated to dryness and dissolved in 5 mL of methanol. The solution was filtered, and the subsequent filtrate was collected for analysis. The Rg1, Re and Rb1 contents in TNK were determined on a Shimadzu Essentia LC-15C HPLC system (Shimadzu, Kyoto, Japan) with a Thermo Syncronis C18 column (150 mm × 4.6 mm, 5 μm, Thermo Scientific, New Jersey, USA). The mobile phase consisted of solvent A (acetonitrile) and solvent B (water). The elution profile for A was 0-35 min, isocratic 19%; 35-55

min, linear gradient of 19-29%; 55-70 min, isocratic 29%; and 70-100 min, linear gradient of isocratic 29-40%. The analysis was performed at 30 ℃, and the absorbance at 203 nm was detected. The Rg1, Re and Rb1 contents were 0.09%, 0.12% and 0.10%, respectively, compared with the standards. Ginsenosides Rg1 (purity >95%), Re (purity >95%) and Rb1 (purity >95%) were purchased from the National Institutes for Food and Drug Control (Beijing, China).

Reference

1. Zhang S, Chen R, Wu H, Wang C (2006) Ginsenoside extraction from Panax quinquefolium L. (American ginseng) root by using ultrahigh pressure. J Pharm Biomed Anal 41: 57-63.
